# Enhanced cellular death in liver and breast cancer cells by dual BET/BRPF1 inhibitors

**DOI:** 10.1002/pro.5191

**Published:** 2024-10-29

**Authors:** Giulia Cazzanelli, Andrea Dalle Vedove, Nicolò Sbardellati, Luca Valer, Amedeo Caflisch, Graziano Lolli

**Affiliations:** ^1^ Department of Cellular, Computational and Integrative Biology—CIBIO University of Trento Trento Italy; ^2^ Department of Biochemistry University of Zürich Zürich Switzerland

**Keywords:** bromodomain inhibitors, cell death, co‐treatment, liver cancer, X‐ray crystallography

## Abstract

The acetylpyrrole scaffold is an acetylated lysine mimic that has been previously explored to develop bromodomain inhibitors. When tested on the hepatoma cell line Huh7 and the breast cancer cell line MDA‐MB‐231, a few compounds in our acetylpyrrole‐thiazole library induced peculiar morphological changes, progressively causing cell death at increasing concentrations. Their evaluation on a panel of human bromodomains revealed concurrent inhibition of BRPF1 and BET bromodomains. To dissect the observed cellular effects, the acetylpyrrole derivatives were compared to JQ1 and GSK6853, chemical probes for the bromodomains of BET and BRPF1, respectively. The appearance of neurite‐like extrusions, accompanied by βIII‐tubulin overexpression, is caused by BET inhibition, with limited effect on cellular viability. Conversely, interference with BRPF1 induces cellular death but not phenotypic alterations. Combined treatment with JQ1 and GSK6853 showed additivity in reducing cellular viability, comparably to the acetylpyrrole‐thiazole‐based BET/BRPF1 inhibitors. In addition, we determined the crystallographic structures of the BRD4 and BRPF1 bromodomains in complex with the acetylpyrrole‐thiazole compounds. The binding modes in the two bromodomains show similar interactions for the acetylpyrrole and different orientations of the moiety that point to the rim of the acetyl‐lysine pocket.

## 1 INTRODUCTION

Bromodomains are protein modules recognizing acetylated lysines (Kac) in histones and other proteins. They are present in 46 human proteins in combination with other functional domains such as methyltransferases, acetylases, and helicases (Filippakopoulos & Knapp, 2014). Interference with Kac binding by bromodomains modified the cellular epigenetic landscape and emerged as a potential therapeutic strategy for various pathologies, especially cancer and inflammation‐related diseases. Among the human bromodomains, the BET (Bromodomain and Extra‐Terminal Domain) sub‐family has been very successfully targeted with small molecules resulting in more than 70 clinical trials (Guo et al., 2023). Lead compounds have been developed for a few other bromodomains outside the BET family (e.g., for BRD9 and SMARCA2, with their efficacy increased by evolving them in targeted degraders exploiting the vulnerability of cancer cells to the loss of these proteins) (Cipriano et al., 2020), while others proved more refractory for the identification of potent and specific inhibitors (Cazzanelli et al., 2023). Dual bromodomain inhibitors also proved efficacious, as observed for the concomitant interference with BET‐CREBBP (CREB‐binding protein) or BET‐BRD7/9 bromodomains (Hügle et al., 2020; Spriano et al., 2020).

Here, we report on the identification of acetylpyrrole derivatives as dual BET‐BRPF1 (bromodomain and PHD finger containing protein 1) hit compounds with affinity values in the single‐digit micromolar range and selective over BRPF2 (aka BRD1) and BRPF3. BRPF1 is a validated target for liver cancer, as demonstrated by both genetic ablation and chemical inhibition (Cheng et al., 2021). When tested on Huh7 hepatocellular carcinoma (HCC) cells, the acetylpyrrole‐based hit compounds caused a definite morphological change, with increased βIII‐tubulin (TUBB3) expression, as previously observed with the BET chemical probe JQ1 in NUT midline carcinoma (Filippakopoulos et al., 2010) and breast cancer (Kanojia et al., 2020), respectively, and here reproduced in HCC, again with the use of JQ1. Furthermore, the acetylpyrrole derivatives promoted cellular death, which is also induced by GSK6853, a BRPF1 chemical probe (Bamborough et al., 2016) here used as a control. Combined treatment with JQ1 and GSK6853 closely reproduces the effects observed with our hit compounds. It also reveals that GSK6853 antagonizes JQ1 with respect to the morphological alteration. Conversely, the two chemical probes act in an additive way when inducing cellular death, with the combined inhibition by JQ1 + GSK6853 (which has similar phenotype as our inhibitors) performing better than the single treatments.

To provide structural support we determined the high‐resolution crystallographic structures of some of the acetylpyrrole‐based hit compounds in complex with BRPF1 and BRD4 bromodomains. The compounds nicely adapt to the different pockets by assuming similar poses with their acetylpyrrole headgroup, but opposing orientations for their tails. Indeed, the tail groups interact with different regions at the rim of the two binding cavities in response to the specific amino acidic substitutions in the two bromodomains.

## 2 RESULTS AND DISCUSSION

**FIGURE 1:**
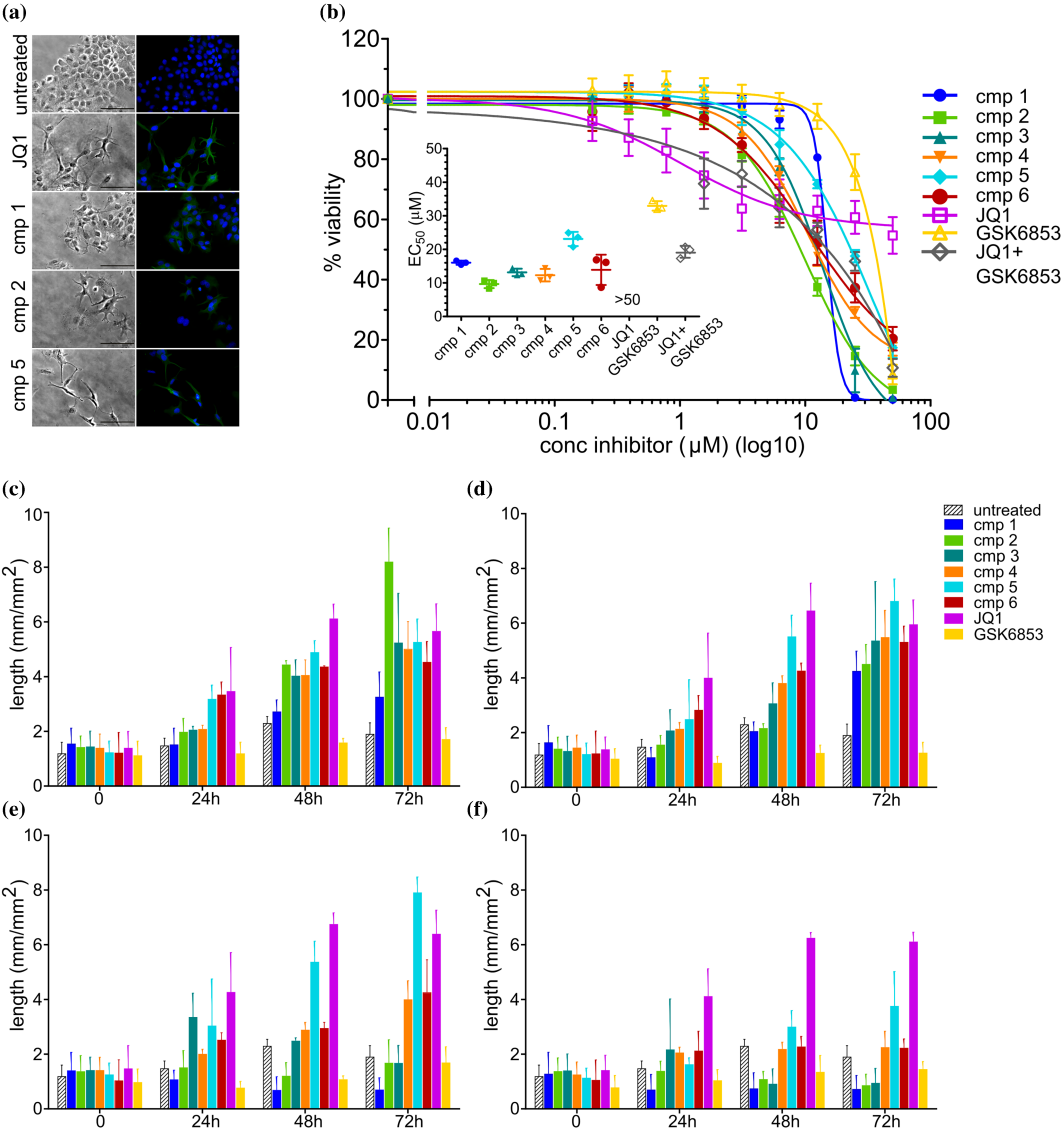
Morphological analysis and viability of Huh7 cell line treated with increasing concentrations of various acetylpyrrole‐thiazole compounds. (a) Huh7 cells were treated for 48 h with the better performing acetylpyrrole‐thiazole compounds (cmp **1** and cmp **2** = 6.25 μM, cmp **5** = 12.5 μM) and with JQ1–1.56 μM—and then immune‐stained to verify the expression of βIII‐tubulin. The bar represents 100 μm. Compounds concentrations were chosen as those determining an appreciable morphological effect but limited impact on cell viability. (b) Viability of Huh7 exposed for 72 h to increasing concentrations of various acetylpyrrole‐thiazole compounds and the reference compounds JQ1 and GSK6853, alone and in combination (1.56 to 50 μM). The insert shows the EC_50_ obtained by interpolating the curves using the log(inhibitor) vs. response—variable slope (four parameters) of GraphPad Prism. For the combination experiment, JQ1 and GSK6853 were varied equally and simultaneously, i.e., each point refers to the indicated concentration for each compound. For both images, each point represents the mean ± SD of 3 independent experiments. (c–f) The graphs show the length of the neurite‐like extrusions caused by treatment with the compounds. JQ1 is always used as positive control. The length was measured using the Incucyte® S3 (Sartorious) software (NeuroTrack analysis). The graphs (c–f) refer to the different concentrations tested: 6.25 μM, 12.5 μM, 25 μM, and 50 μM, respectively. The bars represent the mean ± SD of three independent experiments.

We explored various acetylpyrrole‐thiazole derivatives in a previous campaign aimed at identifying inhibitors for the BAZ2A bromodomain (Dalle Vedove et al., 2022). Interference with this bromodomain was shown to affect stemness and metastatic potential of cancer cell lines with minor effects on proliferation and no morphological alterations (Cazzanelli et al., 2023; Gu et al., 2015; Peña‐Hernández et al., 2021). However, a small subset of our acetylpyrrole‐thiazole compounds (Table 1) induced dramatic alterations in the cellular shape when tested on Huh7 hepatocellular carcinoma cells, suggesting off‐target activities. Upon treatment, cells adopted a stellate morphology with long neurite‐like extrusions, variably accompanied by growth arrest and cellular death (Figure 1 and Figure S1). Similar effects were also observed on the triple‐negative breast cancer MDA‐MB‐231 cell line (Movies [Link], [Link]).

**TABLE 1:** Chemical structure and antiproliferative activity of compounds (CMP) 1–6.

CMP	Structure	EC_50_ (μM)	CMP	Structure	EC_50_ (μM)
1	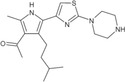	16.0 ± 1.5	5	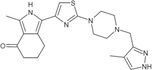	23.1 ± 5.3
2	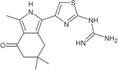	9.6 ± 3.0	6	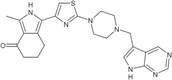	13.9 ± 8.1
3	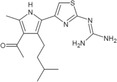	13.1 ± 2.8	JQ1	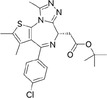	>50
4	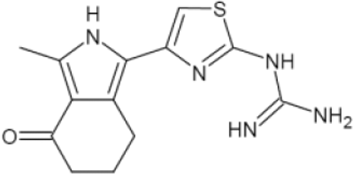	12.3 ± 4.6	GSK6853	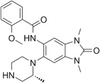	32.9 ± 3.5
			JQ1 + GSK6853^a^		17.7 ± 3.8

^a^ Each compound used simultaneously at equal concentrations. EC50s of JQ1 at fixed GSK6853 concentrations and of GSK6853 at fixed JQ1 concentrations are reported in Tables S1 and S2.

Compound **1** was tested on a panel of 40 bromodomains showing binding to BRPF1 and BET bromodomains with affinity in the very low micromolar range (e.g., 1.4 μM and 3.4 μM for BRPF1 and BRD4, respectively, Table S3 and Figure S2). To dissect the observed cellular effects as determined by BET or BRPF1 inhibition, we compared our inhibitors with JQ1 and GSK6853, chemical probes for BET and BRPF1, respectively (Bamborough et al., 2016; Filippakopoulos et al., 2010). Treatment with JQ1 reproduces the morphological change observed with compounds **1–6**, but with limited cellular death (Table 1, Figure 1, Figure S1). On the contrary, GSK6853 induces substantial cellular death at the highest concentrations tested, without affecting cellular shape. Treatment with compounds **1–6** fully agrees with the expected sequential effects of BET and BRPF1 inhibition. At low micromolar concentrations, the phenotypic change with limited cellular death closely reproduces the JQ1 effects, in accordance with an effective BET bromodomains inhibition. This is also confirmed by TUBB3 overexpression, a known effect associated with JQ1 treatment (Filippakopoulos et al., 2010; Kanojia et al., 2020) and here reproduced with our compounds (Figure 1a, Figures S3 and S4). Instead, compounds **1–6** act almost identically to GSK6853 at the highest concentration tested with exacerbated cellular mortality. At intermediate concentrations, both morphological alterations and cell death are significant, in agreement with a concomitant BET/BRPF1 inhibition. Indeed, co‐treatment with JQ1 and GSK6853 closely reproduces the effects observed with compounds **1–6** (Table 1 and Figure 1b).

**FIGURE 2:**
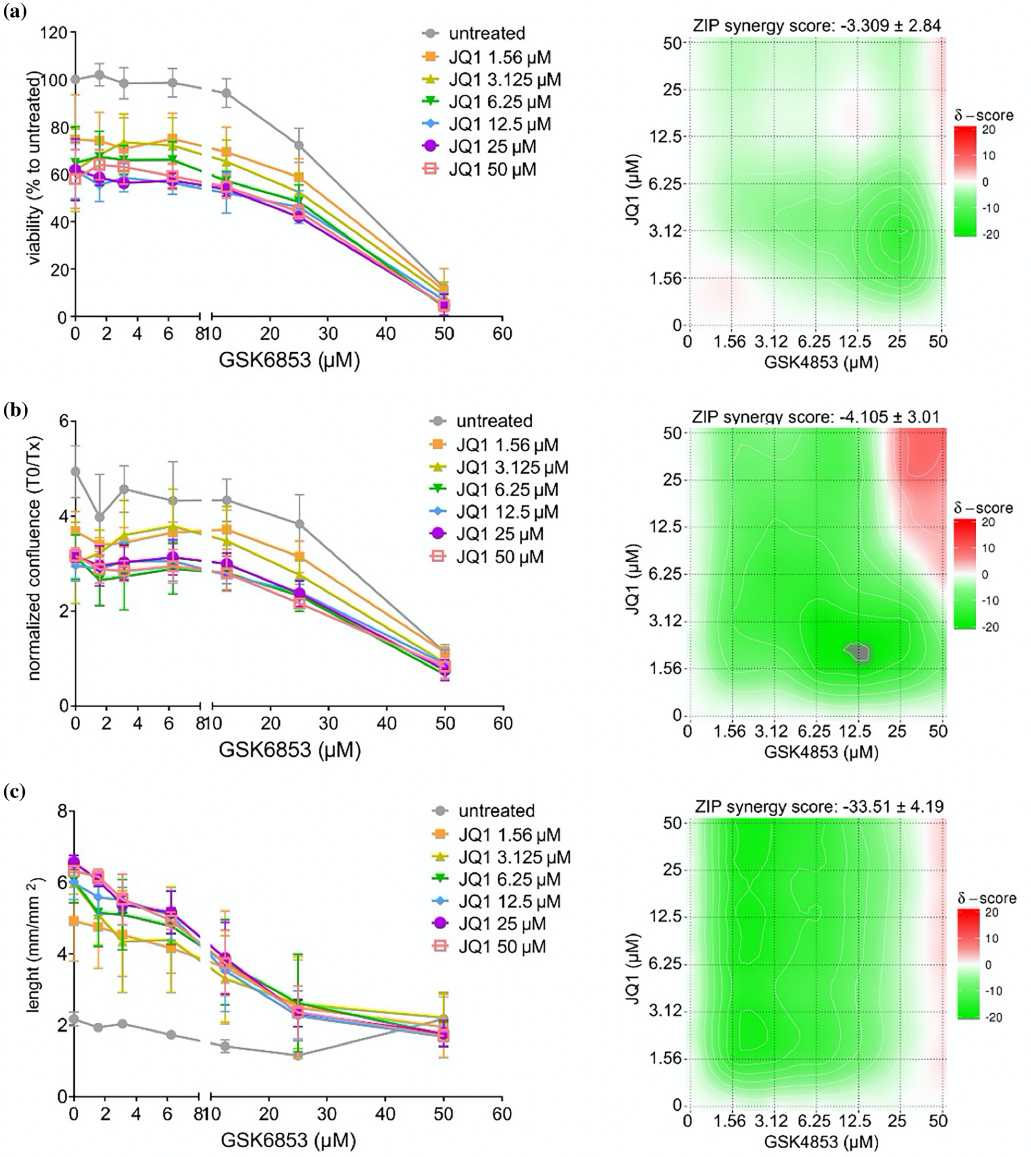
Combined treatment with JQ1 and GSK6853. Huh7 were treated for 72 h with JQ1 and GSK6853 in a combinatorial concentration matrix. The viability (a) and the confluence (b) were measured at the end of the treatment period, while the extrusions length was measured, as previously described, after 48 h (c). The results were plotted in graphs (on the left) where differently colored lines represent different JQ1 concentrations. The combinatorial effect of the two compounds was tested using SynergyFinder (Ianevski et al., 2022) and the results are shown in synergy maps (on the right); synergy score: <−10 = antagonist, from −10 to 10 = additive, larger than 10 = synergistic. The graph bars represent the mean ± SD of three independent experiments, and the synergy maps were obtained using the results of the same three independent experiments.

We then analyzed the reciprocal influence of JQ1 and GSK6853 through a co‐treatment matrix. Regarding cellular viability and growth, JQ1 and GSK6853 show additive, but not synergistic, effects suggesting independent mechanisms (Figure 2a,b). An antagonist effect is instead evident in the cellular morphology, with neurite‐like extrusion disappearing with increasing concentrations of GSK6853 (Figure 2c); again, this reproduces the effect observed with compounds **1–6** (Figure S5).

Cellular BRD4 engagement was confirmed for compound **5** by NanoBRET with EC50 = 65 μM (Figure S6). Compound **5** was chosen as the one less interfering with the assay, i.e., with very low absorbance at 460 and 600 nm compared with most of the others characterized by a dark red color, e.g., compound **1**. A similar assay is not currently available for BRPF1.

**FIGURE 3:**
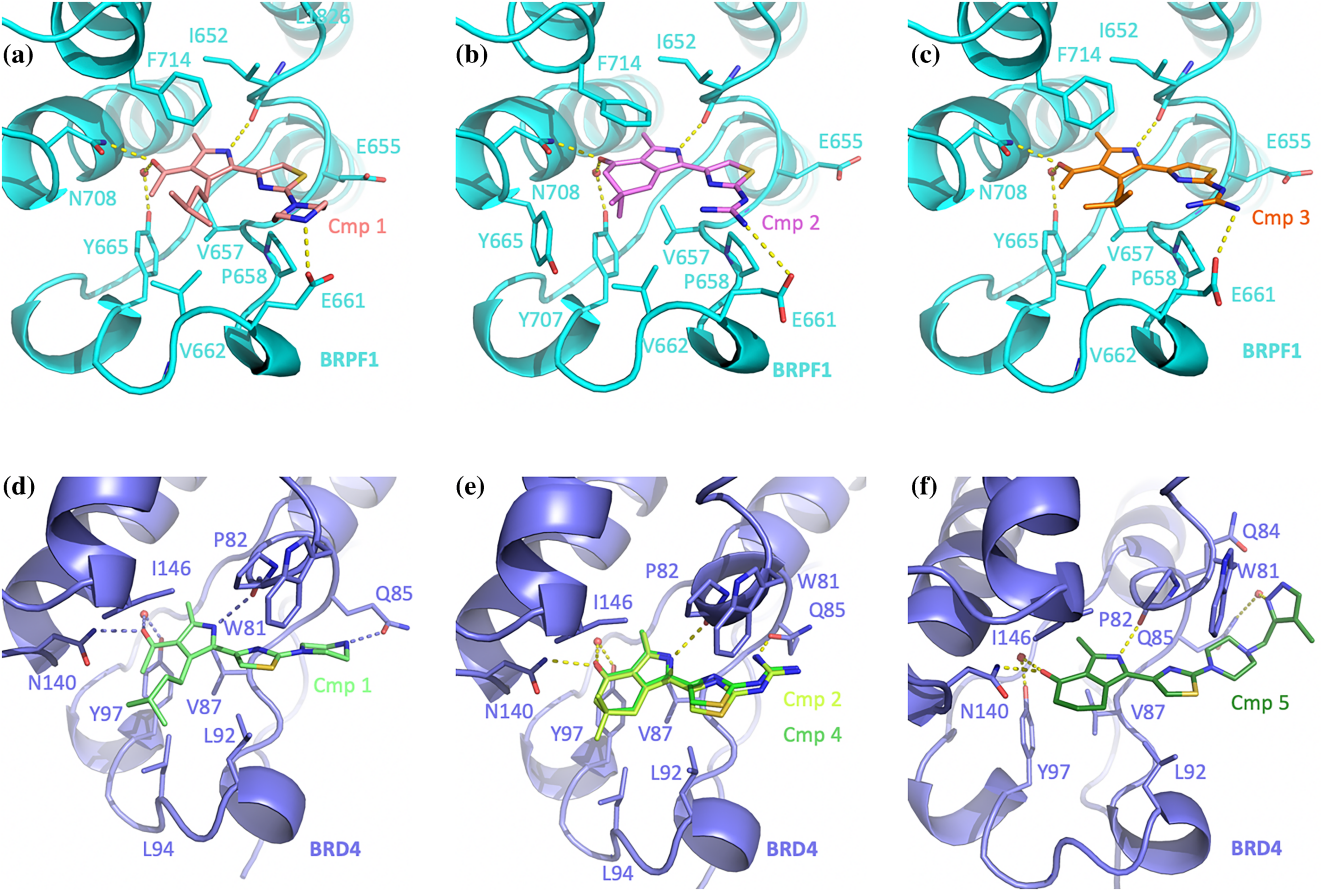
Crystallographic structures of BRPF1 (a–c, cyan) and BRD4 (d–f, slate) in complex with selected inhibitors. Interacting amino acids and inhibitors are shown as sticks, H‐bonds, and ionic interactions as yellow‐dashed lines, and relevant water molecules as red spheres.

Crystallographic structures were determined for compounds **1**, **2**, and **3** in complex with the BRPF1 bromodomain (Figure 3a–c). The common pyrrole ring is sandwiched between side chains of Ile652 and Phe714 on one side and Val657 on the other, also donating a H‐bond to the Ile652 main chain carbonyl. The pyrrole 2‐methyl substituent occupies the same hydrophobic region hosting the Kac methyl group, while the carbonyl in position 3 anchors the oxygen to Asn708 and Tyr665 side chains through hydrogen bonds (the last one being water‐mediated). The thiazole ring sits on the Pro658 (relevant for selectivity over BRPF2, aka BRD1, and BRPF3, Figure S7) side chain, being also in hydrophobic contact with Ile652. The piperazine ring of compound **1** and the guanidinium group in compounds **2** and **3** form a salt bridge with Glu661 side chain. Interestingly, Glu655 could have also been reached by the compounds charged groups subordinately to a 180° flip of the thiazole ring. Nonetheless, the excellent electron density does not allow suspecting of a double binding mode and instead indicates an absolute preference for Glu661. The acetylpyrrole headgroup bears an isopentyl substituent in compounds **1** and **3**, establishing additional contact with side chains of Phe714, Val662, and the hydrocarbon region of Glu661 (Figure 3a,c). The 6,6‐dimethyl‐5,7‐dihydroisoindolone of compound **2** (Figure 3b) induces instead a rotation of Phe714 side chain now stacking almost parallel to the inhibitor; at the same time, a methyl group nicely fits a small hollow surrounded by Val657, Val662, Tyr665, and Tyr707.

The binding mode of compounds **1**, **2**, **4**, and **5** to the first bromodomain of BRD4 was also defined by crystallography (Figure 3d–f). The pyrrole ring is sandwiched between side chains of Ile146 and Val87 forming a hydrogen bond with Pro82 carbonyl oxygen. The 2‐methyl substituent occupies the usual hydrophobic region reserved for the Kac methyl group; hydrogen bonding of the 3‐carbonyl with Asn140 and Tyr97 side chains are also preserved (the last one again water‐mediated). The thiazole ring is squeezed between the side chains of Trp81 and Pro82 on one side and Leu92 on the other. The guanidinium group in compounds **2** and **4** (Figure 3e) interacts with Gln85 side chain (relevant for selectivity on BRD2, BRD3, and BRD4 over BRDT, Figure S8), which maintains the pm0 rotameric orientation (χ_1_ 75°, χ_2_–75°) observed in the apo BRD4 structure (PDB 2OSS). The more protruding piperazine ring of compound **1** induces Gln85 to assume in part the mt‐30 conformer (χ_1_–67°, χ_2_ 177°), establishing a hydrogen bond with its side chain (Figure 3d); notably, however, the electron density sensibly degrades toward the tip of the piperazine ring, indicating flexibility for this region and the associated interaction with Gln85. The extra methylpyrazole group in compound **5** imposes instead the mm‐40 rotameric orientation (χ_1_–66°, χ_2_–60°) to Gln85 forming a water‐bridged hydrogen bond with its side chain (Figure 3f); additional contacts are evident with Trp81 and Gln84. The bulkier headgroups of compounds **1** and **2** extend their van der Waals contacts to Leu94.

**FIGURE 4:**
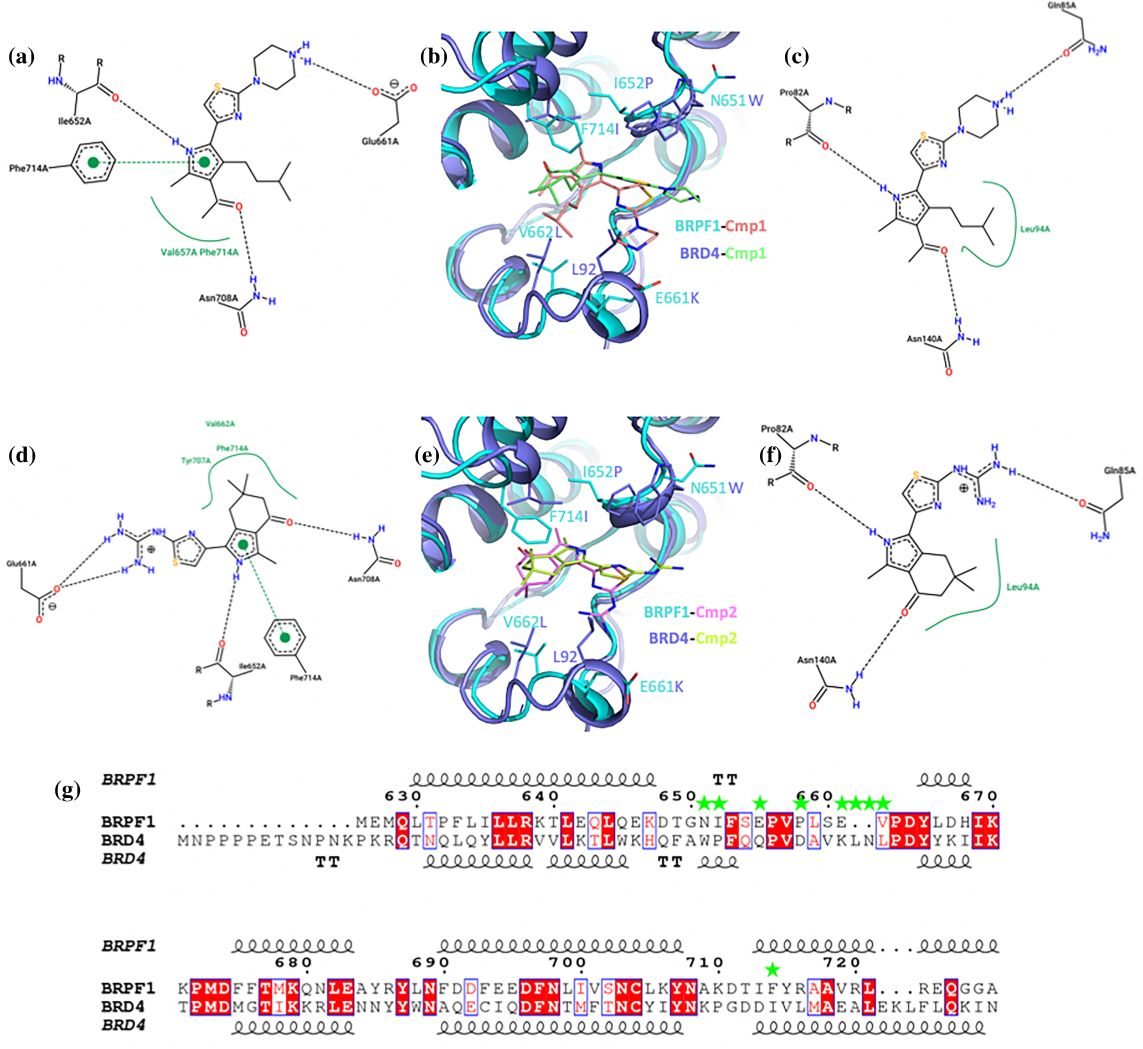
Comparison of binding modes in BRPF1 and BRD4 for compounds **1** and **2**. Schematic diagrams for the interactions of compound **1** with BRPF1 (a) and BRD4 (c) and their superposed crystallographic poses (b, BRPF1 in cyan and BRD4 in slate). Schematic diagrams for the interactions of compound **2** with BRPF1 (d) and BRD4 (f) and their superposed crystallographic poses (e, color code as above). (g) Structural alignment of the two bromodomains. Green stars identify amino acidic changes determining the different binding poses of the reported inhibitors in BRPF1 and BRD4.

Comparison of the binding poses of compounds **1** and **2** in the BRPF1 and BRD4 bromodomains (Figure 4) allows spotting a slightly different tilting of the pyrrole ring mainly dictated by the substitutions of Val662 and Phe714 in BRPF1 with Leu94 and Ile146 in BRD4, also imposing more divergent orientations to the pyrrole C4‐substituents. The most striking difference resides, however, in the almost 180° rotation of the tail moiety adapting to the increasing aminoacidic changes in the two bromodomains when moving toward the tip of the binding pocket. The thiazole ring stacks perpendicularly to Pro658 of the ZA loop in BRPF1 (Asp88 in BRD4), while interacting with Trp81 and Pro82 of the WPF shelf in BRD4 (Asn651 and Ile652 in BRPF1). The charged guanidinium or piperazine terminal groups form salt bridges with Glu661 in BRPF1 (Lys91 in BRD4); the same orientation is also impeded in BRD4 by the ^92^LN^93^ insertion clashing with the inhibitor. Instead, the charged tails interact with BRD4 Gln85, also forming a π‐cation interaction with Trp81, although with suboptimal geometry (Marshall et al., 2009). The similar affinity of compound **1** for BRPF1 and BET bromodomains can then be ascribed to its adaptation to the different pockets, returning partially dissimilar interactions but with overall comparable energetic contributions. Such adaptation is preserved in compound **2** (Figure 4) and possibly in the other inhibitors here described and maintaining their BET‐ or BRPF1‐specific pose (Figure 3).

## 3 CONCLUSIONS

We report on the identification of acetylpyrrole‐thiazole derivatives as dual BET/BRPF1 bromodomain inhibitors that reproduce the cellular effects observed by the combined treatment with BET and BRPF1 chemical probes. More specifically, concurrent interference with BET/BRPF1 bromodomains revealed superior to the treatments with the single chemical probes for BET (JQ1) and BRPF1 (GSK6853) in inducing Huh7 growth inhibition and cellular death, respectively. The observed versatility in the binding mode of the acetylpyrrole‐thiazole‐based compounds provides definitive evidence of target engagement and corroborates their similar affinities for the bromodomains of BRD4 and BRPF1.

## 4 EXPERIMENTAL PROCEDURES

### 4.1 Chemicals and BROMOscan assay

Compounds **1–6** were purchased from either Enamine Ltd. or SIA Chemspace with a purity >95%. JQ1 and GSK6853 were obtained from CliniSciences. BROMOscan was performed by Eurofins DiscoverX Corporation, a detailed description of the assay technology is presented in ref. (Fabian et al., 2005).

### 4.2 Cell culture

The hepatocarcinoma cell line Huh7 and the breast cancer cell line MDA‐MB‐231 were cultured in DMEM medium. The medium contained 10% FBS, 1% penicillin–streptomycin, 2 mM L‐glutamine. All cell lines were regularly tested for mycoplasma contamination.

### 4.3 Cell viability and Incucyte growth curves

Huh7 and MDA‐MB‐231 cells were counted with a hemocytometer and seeded in triplicates in 96‐well plates (2.5 × 10^3^ and 3.0 × 10^3^ cells/well, respectively) and allowed to adhere overnight at 37°C, 5% CO_2_. The following day, the medium was replaced by adding either the carrier (0.1% DMSO) or the inhibitor (0.19–0.39–0.78–1.56–3.125–6.25–12.5–25–50 μM acetylpyrrole‐thiazole compounds and 1.56–3.125–6.25–12.5–25–50 μM JQ1 and GSK68535) resuspended in the same amount of carrier. In the case of double treatment, the two molecules were added simultaneously, and the amount of carrier in the untreated sample was adjusted to match that in the treated one.

Cell growth and morphology alterations were monitored by imaging the cells (4 fields/well, 10× magnification) with the Incucyte® S3 (Sartorious) every 24 h for Huh7 and every 2 h for MDA‐MD‐231. Huh7 cells viability was measured after 72 h by adding 10% resazurin sodium salt solution (0.03 mg/mL powder from Sigma‐Aldrich dissolved in PBS) directly into each well and incubating the cells for 4 h at 37°C, 5% CO_2_. The 570/600 nm fluorescence was measured using Varioskan™ LUX (Thermo Scientific™) plate reader. The viability was calculated as percentage compared to the sample treated with the carrier only, considered 100% viable. The log(inhibitor) vs. response—variable slope (four parameters) model of the GraphPad Prism software was used to determine the EC_50_ of each replicate, which was then averaged.

Cell confluence and morphology alterations were analyzed using the Incucyte® S3 (Sartorious) software, specifically the Basic Analyzer tool for confluence and the NeuroTrack for measuring the length of the cellular processes.

### 4.4 Western blot and immunofluorescence

Cells were counted and seeded at 25.000 cells/well in a 12‐well plate. For immunofluorescence, the cells were seeded on a coverslip.

For western blot, total protein content was extracted from cells treated for 72 h with different concentrations of cmp **1**, cmp **2**, cmp **5**, and JQ1 (6.25 μM or 12.5 μM–6.25 μM–12.5 μM and 1.56 μM, respectively). Cells were lysed using RIPA buffer, and protein concentration was determined by Bradford Reagent (Sigma‐Aldrich), according to the manufacturer's instructions. 30 μg of proteins per sample in Laemmli Buffer 4× were run in a precast stain‐free gel (nUView Tris‐Glycine Precast Gels, NuSep) until complete separation. Proteins were then blotted on a PVDF (polyvinylidene difluoride) membrane, which was blocked in 5% skimmed milk and 0.1% Tween 20 in TBS for 2 h at room temperature and then treated with the appropriate primary (4°C overnight) and secondary (2 h at room temperature) antibodies and revealed by chemiluminescence (ECL Amersham). Used antibodies were anti‐βIIItubulin 1:10000 (Proteintech) and secondary anti‐mouse 1:4000 (Santa Cruz Biotechnology). Densitometric analysis was performed using ImageLab (Biorad) software, and protein expression levels were normalized to the total protein level imaged in the stain‐free membrane.

For immunofluorescence, the cells were seeded and treated the same way as for western blot. After 48 h, they were fixed with 4% PFA for 12 min, rinsed with PBS, and incubated first with anti‐βIIItubulin 1:500 (Proteintech) overnight at 4°C and then with anti‐mouse AlexaFluor™ 488 1:1000 (Invitrogen) for 2 h at room temperature. The images were obtained using a Leica DM IL microscope equipped with a Leica DFC450C digital camera, with a 40× magnification.

### 4.5 NanoBRET cellular target engagement assay

NanoBRET assay was performed by Reaction Biology. Assay reagents were all purchased from Promega. HEK293 cells were transiently transfected with NanoLuc®‐BRD4 FL Fusion Vector DNA by FuGENE HD Transfection Reagent. Testing compounds were delivered into a 384 well assay plate by Echo 550 liquid handler (Labcyte Inc., Sunnyvale, CA). Transfected cells were harvested and mixed with NanoBRET® Intracellular TE BET BRD Tracer and dispensed into 384 well plates. Plates were incubated at 37°C in 5% CO_2_ cell culture incubator for 1 hour. NanoBRET® Nano‐Glo® Substrate plus Extracellular NanoLuc® Inhibitor Solution was added into the wells of the assay plate and incubated for 20 min at room temperature. The donor emission wavelength (460 nm) and acceptor emission wavelength (600 nm) were measured on an Envision 2104 Multilabel Reader (PerkinElmer, Santa Clara, CA). BRET Ratio was calculated. BRET Ratio = [(Acceptor sample)/(Donor sample)]—[(Acceptor no‐tracer control)/(Donor no‐tracer control)]. The IC50 curves were plotted and IC50 values were calculated using the GraphPad Prism 4 program based on a sigmoidal dose–response equation.

### 4.6 Protein purification and X‐ray crystallography

BRPF1 and BRD4 bromodomains were produced as previously described (Marchand et al., 2017; Unzue et al., 2016). BRD4 crystals were obtained in the conditions previously reported (Lolli & Battistutta, 2013). BRPF1 was crystallized at 4°C in either 0.1 M sodium citrate pH 5.5 + 0.18 M NaNO_3_ + 30% PEG3350 or 0.1 M HEPES pH 7.5 + 0.18 M NaNO_3_ + 30% PEG3350 + 5% ethylene glycol. Compounds (10 mM) were soaked in both BRD4 and BRPF1 crystals over 48 hours in cryoprotective solutions (crystallization solutions +20% ethylene glycol). DMSO was avoided or kept to a minimum (0.1%) as it can compete for binding to the bromodomains Kac pocket (Lolli & Battistutta, 2013). Compounds were not fully soluble in the tested conditions; structures not here reported showed partial occupancy at the inhibitor site with complicated densities deriving from combination of the holo and apo forms. Compound **6** was not tested as the least soluble one.

Diffraction data were collected at the Elettra Synchrotron Light Source (Trieste, Italy), XRD2 beamline. Data were processed and structures were solved as described elsewhere (Spiliotopoulos et al., 2017). Data collection and refinement statistics are reported in Tables S4 and S5. Electron densities (2F_o_‐F_c_ polder OMIT map (Liebschner et al., 2017)) for the bound inhibitors are shown in Figure S9.

### 4.7 Accession codes

Structures were deposited to the PDB with accession numbers 8QAN (BRD4/cmp **1**), 8QAP (BRD4/cmp **2**), 8QAL (BRD4/cmp **4**), 8QAR (BRD4/cmp **5**), 8QB2 (BRPF1/cmp **1**), 8QB0 (BRPF1/cmp **2**), 8QAZ (BRPF1/cmp **3**). Atomic coordinates and experimental data will be released upon article publication.

## AUTHOR CONTRIBUTIONS

**Giulia Cazzanelli:** Investigation; writing – original draft; conceptualization; formal analysis. **Andrea Dalle Vedove:** Investigation; formal analysis. **Nicolò Sbardellati:** Investigation. **Luca Valer:** Investigation. **Amedeo Caflisch:** Conceptualization; funding acquisition; writing – review and editing; resources. **Graziano Lolli:** Conceptualization; funding acquisition; writing – original draft; writing – review and editing; formal analysis; supervision; resources.

## CONFLICT OF INTEREST STATEMENT

The authors declare no competing financial interest.

## Supporting information


**Data S1:** Supplementary Tables: JQ1 EC_50_s in co‐treatment at fixed concentrations of GSK6853; GSK6853 EC_50_s in co‐treatment at fixed concentrations of JQ1; BROMOscan measured affinities for compound **1**; X‐ray data collection and refinement statistics for all determined crystallographic structures.
Supplementary Figures: Morphological alterations in Huh7 cells; BROMOscan dose/response curves; WB quantifications; dose/response curves for extrusions length; cellular target engagement by NanoBRET; structural comparison of BRPF and BET family members; F_o_‐F_c_ electron density maps for compounds in complex with the BRD4 and BRPF1 bromodomains.

**Movie S1.** Untreated: time‐lapse movie of untreated MDA‐MB‐231 cells.

**Movie S2.** cmp1: time‐lapse movie of MDA‐MB‐231 cells treated with compound 1.

**Movie S3.** cmp2: time‐lapse movie of MDA‐MB‐231 cells treated with compound 2.

**Movie S4.** cmp3: time‐lapse movie of MDA‐MB‐231 cells treated with compound 3.

**Movie S5.** cmp4: time‐lapse movie of MDA‐MB‐231 cells treated with compound 4.

**Movie S6.** cmp5: time‐lapse movie of MDA‐MB‐231 cells treated with compound 5.

**Movie S7.** cmp6: time‐lapse movie of MDA‐MB‐231 cells treated with compound 6.
