# Supplementary material for: Enhanced cellular death in liver and breast cancer cells by dual BET/BRPF1 inhibitors
Source: Protein Sci. 2024 Oct 29;33(11):e5191. doi: 10.1002/pro.5191 (PMC11521936; doi:10.1002/pro.5191)
Supplement: Supplementary file 1 — Data S1: Supplementary Tables: JQ1 EC50s in co‐treatment at fixed concentrations of GSK6853; GSK6853 EC50s in co‐treatment at fixed concentrations of JQ1; BROMOscan measured affinities for compound 1; X‐ray data collection and refinement statistics for all determined crystallographic structures. Supplementary Figures: Morphological alterations in Huh7 cells; BROMOscan dose/response curves; WB quantifications; dose/response curves for extrusions length; cellular target engagement by NanoBRET; structural comparison of BRPF and BET family members; Fo‐Fc electron density maps for compounds in complex with the BRD4 and BRPF1 bromodomains. [file PRO-33-e5191-s001.pdf]

SUPPLEMENTARY MATERIAL

Enhanced cellular death in liver and breast cancer cells by dual BET/BRPF1 inhibitors

Giulia Cazzanelli[1*], Andrea Dalle Vedove[1], Nicolò Sbardellati[1], Luca Valer[1], Amedeo Caflisch[2] and Graziano Lolli[1*]

Author Affiliations:

[1]Department of Cellular, Computational and Integrative Biology - CIBIO, University of Trento, via Sommarive 9, 38123 Povo - Trento, Italy

[2]Department of Biochemistry, University of Zürich, Winterthurerstrasse 190, CH-8057 Zürich, Switzerland

**Correspondence to Giulia Cazzanelli and Graziano Lolli*: giulia.cazzanelli@unitn.it; graziano.lolli@unitn.it.

Supplementary Table 1. JQ1 EC_{50}s in co-treatment at fixed concentrations of GSK6853.

**[GSK6853] (µM)**	0	1.56	3.12	6.25	12.5	25	50
**JQ1 EC_{50} (µM)**	> 50	> 50	> 50	> 50	> 50	6.6 ± 6.0	n.d.*

*excessive death caused by GSK6853.

Supplementary Table 2. GSK6853 EC_{50}s in co-treatment at fixed concentrations of JQ1.

**[JQ1] (µM)**	0	1.56	3.12	6.25	12.5	25	50
**GSK6853 EC_{50} (µM)**	32.9 ± 3.5	28.7 ± 6.1	25.8 ± 6.0	22.8 ± 4.8	16.8 ± 12.1	16.9 ± 2.4	19.0 ± 5.4

Supplementary Table 3. Binding affinities for compound 1 as measured by BROMOscan

Gene Symbol	Kd (µM)
ATAD2A	>100
ATAD2B	>100
BAZ2A	72 ± 10
BAZ2B	>100
BRD1	>100
BRD2(1)	9.7 ± 1.4
BRD2(1,2)	6.1 ± 1.1
BRD2(2)	7.9 ± 0.2
BRD3(1)	6.1 ± 0.3
BRD3(1,2)	4.2 ± 0.2
BRD3(2)	4.8 ± 1.5
BRD4(1)	5.2 ± 0.2
BRD4(1,2)	3.4 ± 0.5
BRD4(2)	4.4 ± 0.3
BRD4(full-length, short iso.)	3.6 ± 0.5
BRD7	12 ± 1
BRD8(1)	>100
BRD8(2)	>100
BRD9	55 ± 2.5
BRDT(1)	14 ± 1.5
BRDT(1,2)	18 ± 2
BRDT(2)	20 ± 0.1
BRPF1	1.4 ± 0.6
BRPF3	>100
CECR2	95 ± 10
CREBBP	51 ± 5
EP300	51 ± 10
FALZ	96 ± 1.5
GCN5L2	>100
PBRM1(2)	>100
PBRM1(5)	>100
PCAF	>100
SMARCA2	>100
SMARCA4	>100
TAF1(2)	>100
TAF1L(2)	>100
TRIM24(bromo)	>100
TRIM24(PHD,Bromo)	>100
TRIM33(PHD,Bromo)	>100
WDRR9(2)	>100

Numbers in parentheses indicate the bromodomain tested for proteins containing multiple bromodomains.

Supplementary Table 4. Data Collection and Refinement Statistics for BRPF1 structures.

	Cmp **1**	Cmp **2**	Cmp **3**
Data Collection			
Space group	$P3_221$	$P3_221$	$P3_221$
Unit-cell parameters (Å, °)	a = 60.49<br>b = 60.49<br>c = 62.23	a = 60.54<br>b = 60.54<br>c = 63.26	a = 60.55<br>b = 60.55<br>c = 62.51
Wavelength (Å)	1.00	1.00	1.00
Resolution (Å)	62.23-1.42<br>(1.45-1.42)	63.26-1.45<br>(1.47-1.45)	62.51-1.40<br>(1.42-1.40)
R_{merge} (%)	4.1 (160.1)	6.2 (115.5)	3.9 (156.7)
R_{meas} (%)	4.2 (164.5)	6.4 (118.7)	4.0 (161.4)
R_{pim} (%)	1.0 (37.7)	1.5 (27.5)	1.0 (38.3)
$<I/\sigma(I)>$	31.0 (2.0)	24.2 (2.9)	33.8 (2.1)
$CC^{1/2}$	1.000 (0.821)	0.998 (0.893)	1.000 (0.768)
Completeness (%)	100.0 (100.0)	100.0 (100.0)	100.0 (100.0)
Multiplicity	18.0 (18.8)	18.1 (18.5)	18.2 (17.6)
Refinement			
Resolution (Å)	52.42-1.42	52.47-1.45	52.48-1.40
R_{work}/R_{free} (%)	16.8/19.6	17.9/20.8	16.3/19.5
R.m.s. deviations			
Bond lengths (Å)	0.008	0.009	0.008
Bond angles (°)	0.93	0.99	0.91
PDB entry	8QB2	8QB0	8QAZ

Supplementary Table 5. Data Collection and Refinement Statistics for BRD4 structures.

	Cmp **1**	Cmp **2**	Cmp **4**	Cmp **5**
Data Collection				
Space group	$P2_12_12_1$	$P2_12_12_1$	$P2_12_12_1$	$P2_12_12_1$
Unit-cell parameters (Å, °)	a = 37.06<br>b = 44.48<br>c = 78.11	a = 37.10<br>b = 44.37<br>c = 78.00	a = 37.21<br>b = 44.67<br>c = 77.73	a = 37.32<br>b = 44.38<br>c = 78.42
Wavelength (Å)	1.00	1.00	1.00	1.00
Resolution (Å)	39.05-1.25<br>(1.27-1.25)	44.37-1.40<br>(1.42-1.40)	38.87-1.30<br>(1.32-1.30)	78.42-1.50<br>(1.53-1.50)
R_{merge} (%)	9.4 (121.5)	10.9 (144.9)	8.3 (80.7)	15.0 (118.7)
R_{meas} (%)	9.8 (126.7)	11.4 (151.1)	8.7 (85.1)	15.6 (124.2)
R_{pim} (%)	2.8 (35.5)	3.3 (42.2)	2.7 (26.6)	4.5 (36.0)
$<I/\sigma(I)>$	14.0 (2.4)	12.5 (2.1)	15.3 (3.6)	9.6 (2.2)
$CC^{1/2}$	0.999 (0.793)	0.998 (0.767)	0.999 (0.857)	0.994 (0.770)
Completeness (%)	98.2 (95.2)	100.0 (100.0)	95.7 (92.3)	100.0 (100.0)
Multiplicity	12.4 (12.4)	12.2 (12.6)	10.0 (10.0)	12.3 (11.7)
Refinement				
Resolution (Å)	39.05-1.25	39.02-1.40	38.87-1.30	39.22-1.50
R_{work}/R_{free} (%)	15.4/17.6	15.1/18.6	14.6/17.1	16.6/20.1
R.m.s. deviations				
Bond lengths (Å)	0.009	0.008	0.008	0.009
Bond angles (°)	1.12	0.94	1.05	1.05
PDB entry	8QAN	8QAP	8QAL	8QAR

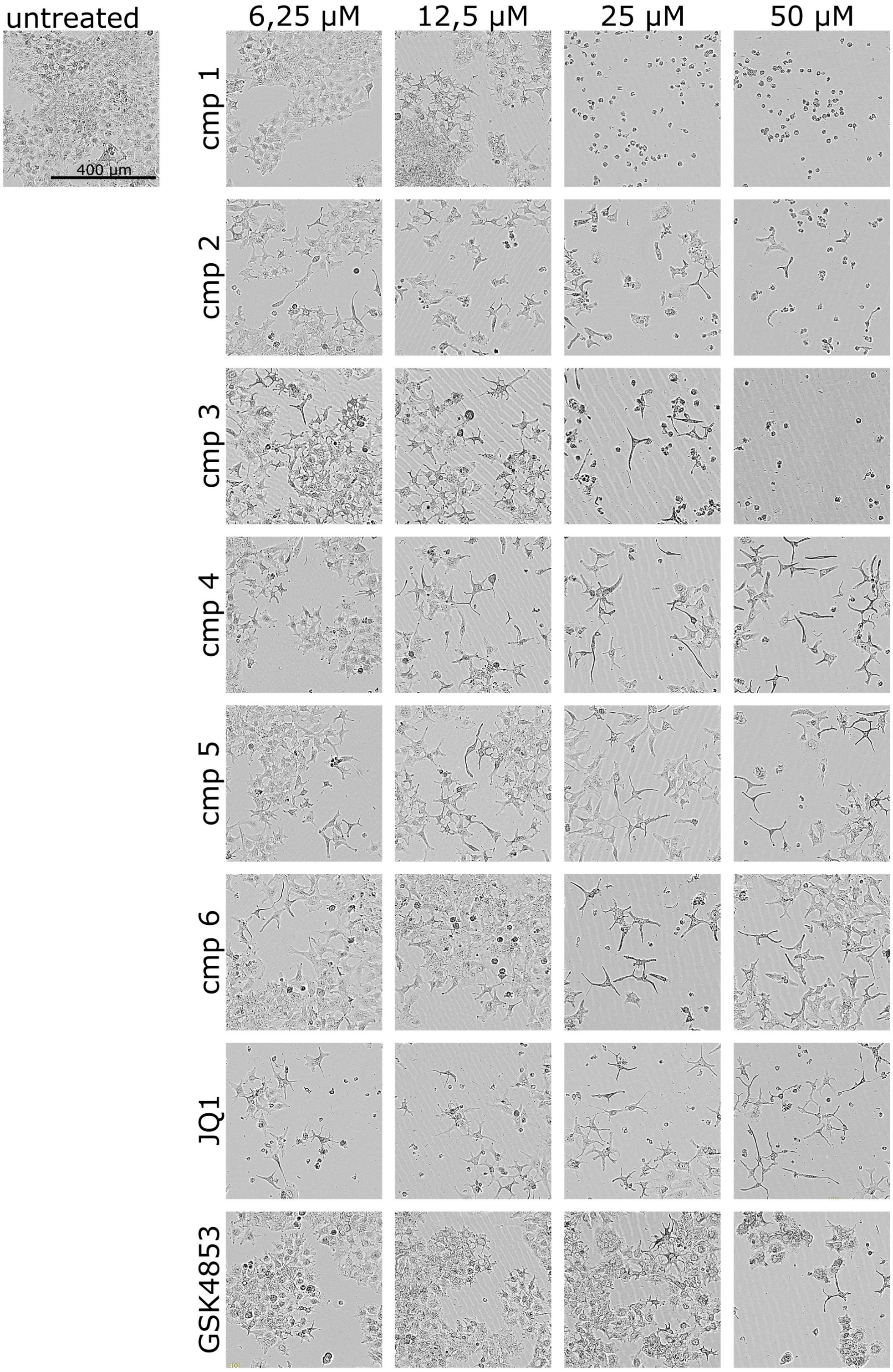

Supplementary Figure 1: morphological changes of Huh7. The pictures show the morphological changes of Huh7 treated for 72h with the acetylpyrrole-thiazole compounds and the reference compounds JQ1 and GSK6853. The concentrations are the same showed in Fig. 1D-E. The images were obtained from the Incucyte® S3 (Sartorious), magnification 10x. The images are representative of one of three independent experiments.

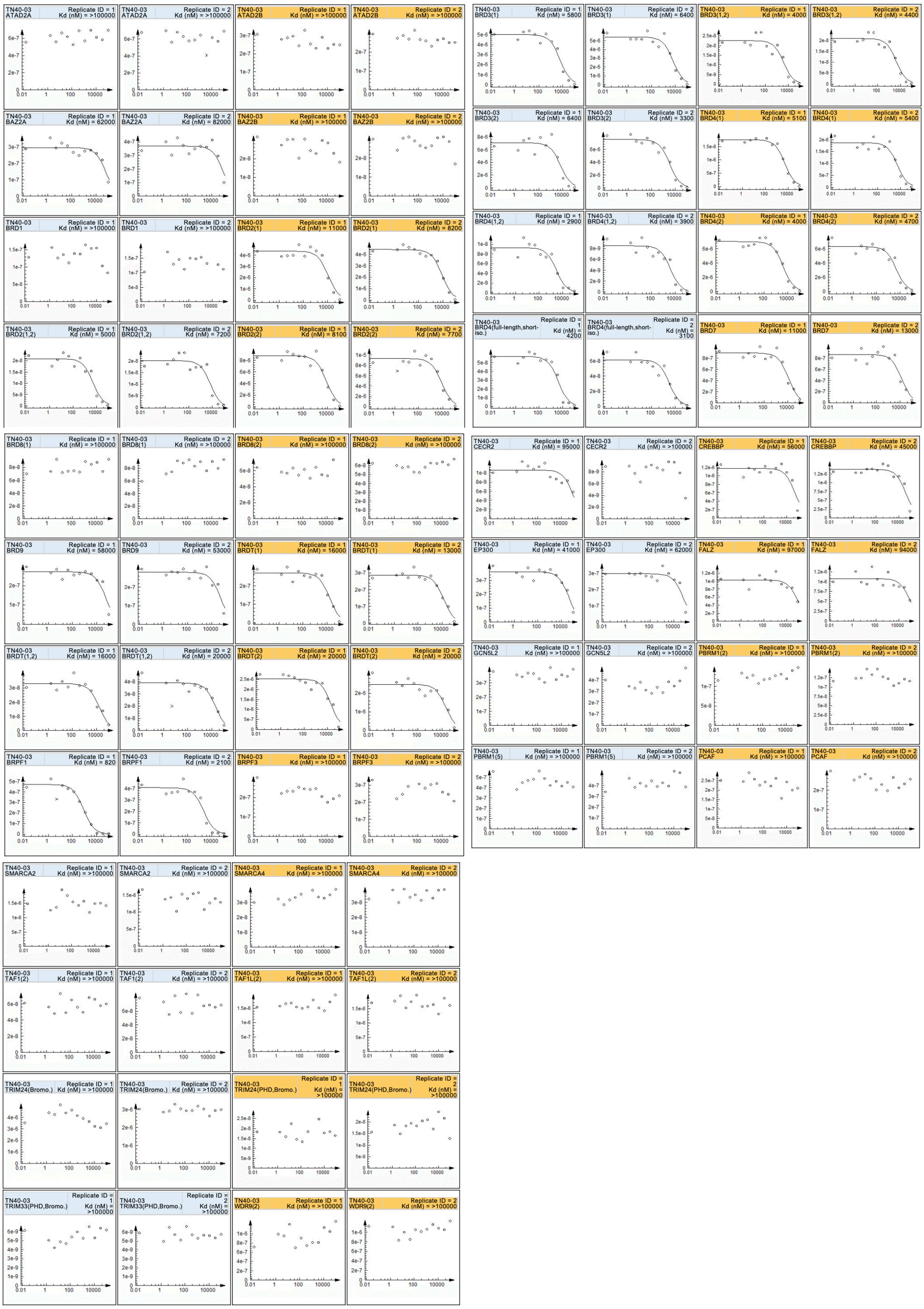

Supplementary Figure 2. BROMOscan binding curves for compound 1. Binding to each bromodomain has been performed in duplicate. Bromodomain tested is indicated in each graph together with calculated K_d. Compound 1 is named TN40-03 in the graphs (internal library name as communicated to Eurofins).

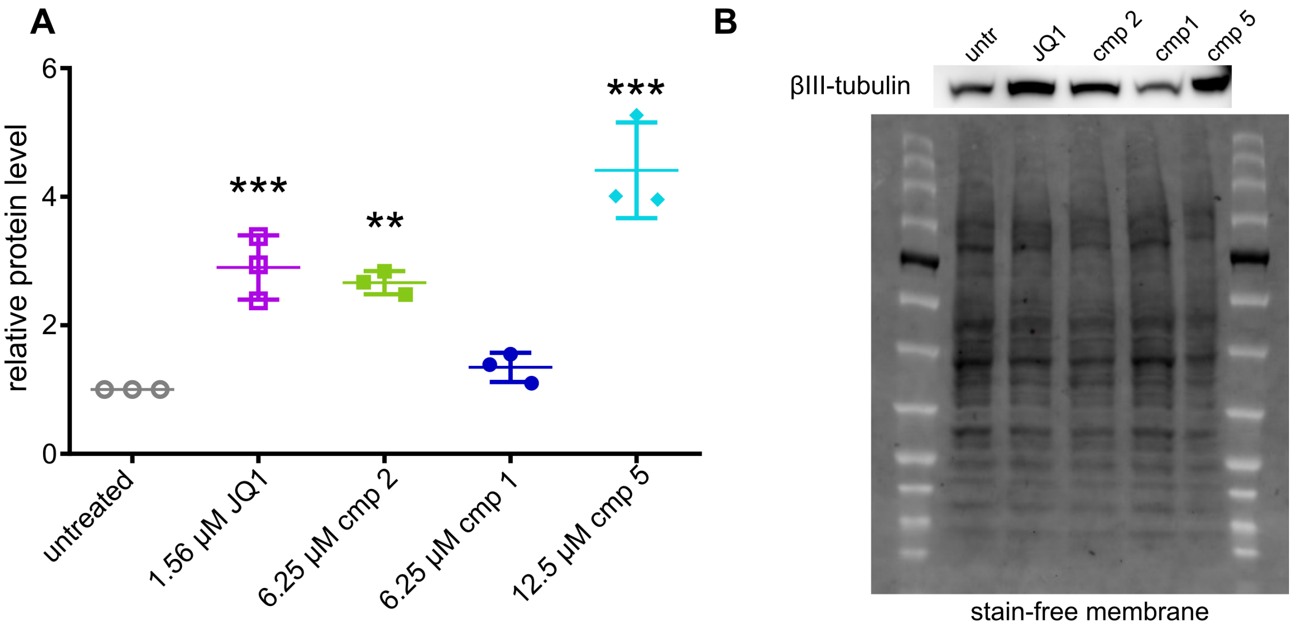

Supplementary Figure 3. Western blot quantification. βIII-tubulin protein level relative to the untreated cells and normalized using the stain free method. The bars represent the mean ± SD of 3 independent experiments. Cells were treated for 48 hours.

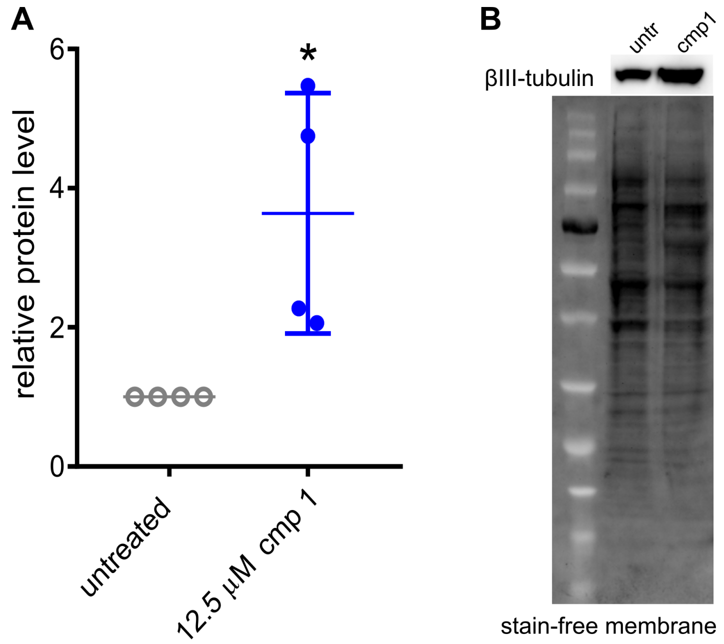

Supplementary Figure 4. Western blot quantification. βIII-tubulin protein level relative to the untreated cells and normalized using the stain free method. The bars represent the mean ± SD of 4 independent experiments. Cells were treated for 72 hours.

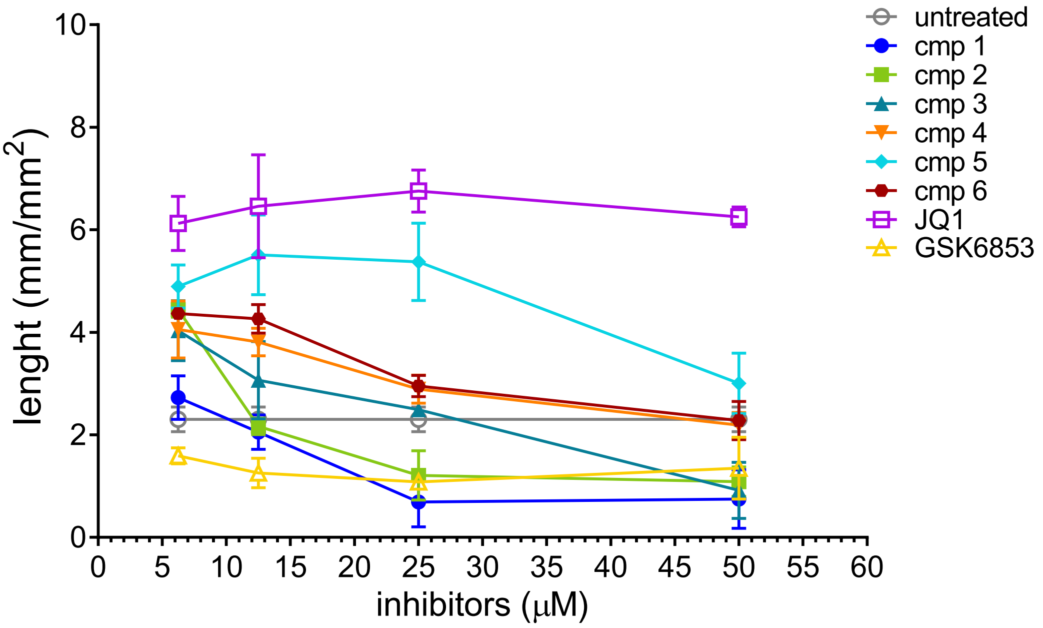

Supplementary Figure 5: Effect of treatment on extrusions length. Huh7 extrusions were measured using the Incucyte® S3 (Sartorious) software (NeuroTrack analysis) after 48h treatment with the indicated compounds. Each point represents the mean ± SD of 3 independent experiments.

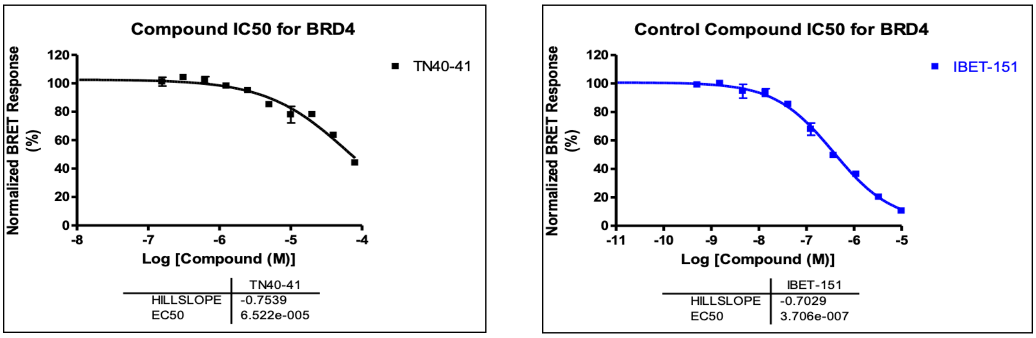

Supplementary Figure 6: Cellular target engagement by NanoBRET. Dose-response curves for compound 5 (black, left) and control compound IBET-151 (blue, right). Compound 5 is named TN40-41 in the graph (internal library name as communicated to Reaction Biology). Experiments were conducted in duplicate.

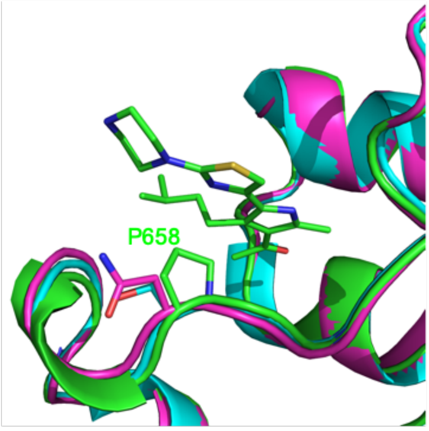


```
BRPF1        MQLTPFLILLRKTLEQLQEKDTGNIFSEPVPLSEVPDYLDHIKKPMDFFTMKQNLEAYRYLNFDDFEEDFNLIVSNCLKY
BRPF2_BRD1    RLTPLTVLLRSVLDQLQDKDPARIFAQPVSLKEVPDYLDHIKHPMDFATMRKRLEAQGYKNLHEFEEDFDLIIDNCMKY
BRPF3          LMPFNVLLRTTLDLLQEKDPAHIFAEPVNLSEVPDYLEFISKPMDFSTMRRKLESHLYRTLEEFEEDFNLIVTNCMKY
               * *: :***..*: **:**...**::** *.******:.*.:**** **::.**:  * .:.:*****:**: **:**

BRPF1          NAKDTIFYRAAVRLREQGGAVLRQARRQAEKM--------
BRPF2_BRD1_    NARDTVFYRAAVRLRDQGGVVLRQARREVDSIGLEEASGM
BRPF3          NAKDTIFHRAAVRLRDLGGAILRHARRQAENIG-------
               **:**:*:*******: **.:**:***:.:.:
```

Supplementary Figure 7: Structural comparison of BRPF family members. Pro658 in BRPF1 (green) allows for a better accommodation of compound 1 with respect to the corresponding amino acids Ser in BRPF2 (cyan) and Asn in BRPF3 (magenta). The aminoacidic substitution is indicated with a green star in the sequence alignment. All other amino acids interacting with the inhibitor are conserved.

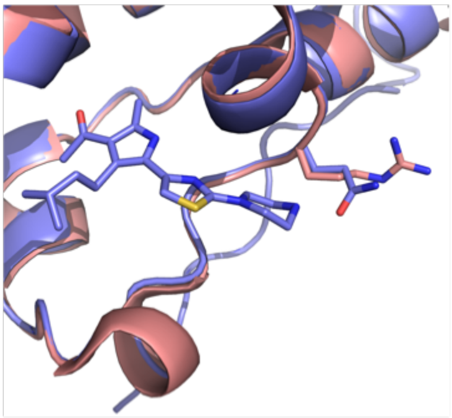

```
BRDT      ----------------TNQLQYLQKVVLKDLWKHSFSWPFQRPVDAVKLQLPDYYTIIKN
BRD4      NPPPPETSNPNKPKRQTNQLQYLLRVVLKTLWKHQFAWPFQQPVDAVKLNLPDYYKIIKT
BRD2      -------------GRVTNQLQYLHKVVMKALWKHQFAWPFRQPVDAVKLGLPDYHKIIKQ
BRD3      -----EVSNPSKPGRKTNQLQYMQNVVVKTLWKHQFAWPFYQPVDAIKLNLPDYHKIIKN
                          ******: .**:* ****.*:*** :****:** ****:.***

BRDT      PMDLNTIKKRLENKYYAKASECIEDFNTMFSNCYLYNKPGDDIVLMAQALEKLFMQKLSQ
BRD4      PMDMGTIKKRLENNYYWNAQECIQDFNTMFTNCYIYNKPGDDIVLMAEALEKLFLQKINE
BRD2      PMDMGTIKRRLENNYYWAASECMQDFNTMFTNCYIYNKPTDDIVLMAQTLEKIFLQKVAS
BRD3      PMDMGTIKKRLENNYYWSASECMQDFNTMFTNCYIYNKPTDDIVLMAQALEKIFLQKVAQ
          ***:.***:****:**  *.**::******:***:**** *******::***:*:**: .
```

Supplementary Figure 8: Structural comparison of BET family members. Gln85 in BRD4 (purple) allows for a better interaction of compound 1 with respect to the corresponding amino acids Arg in BRDT (pink). The aminoacidic substitution is indicated with a red star in the sequence alignment. All other amino acids interacting with the inhibitor are conserved (green stars).

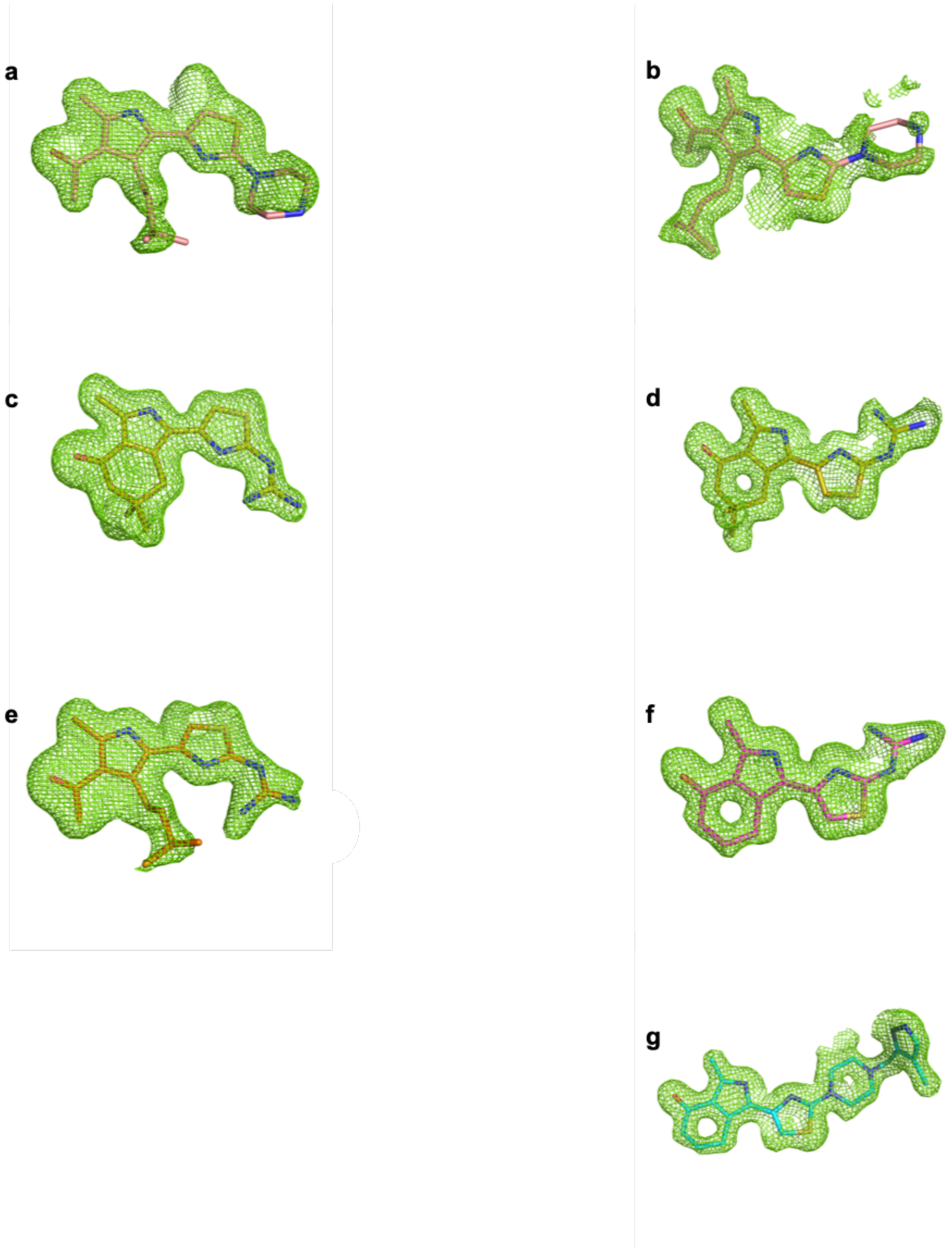

Supplementary Figure 9: F_o-F_c polder OMIT maps contoured at 3 σ for: a) cmp 1 bound to BRPF1, b) cmp 1 bound to BRD4, c) cmp 2 bound to BRPF1, d) cmp 2 bound to BRD4, e) cmp 3 bound to BRPF1, f) cmp 4 bound to BRD4 and g) cmp 5 bound to BRD4.
